# Massic Activity Ratios of the NBS/NIST Tritiated-Water Standards Issued Between 1954 and 1999

**DOI:** 10.6028/jres.105.042

**Published:** 2000-08-01

**Authors:** L. L. Lucas

**Affiliations:** National Institute of Standards and Technology, Gaithersburg, MD 20899-8462 U.S.A

**Keywords:** hydrogen-3, massic activity, NBS, NIST, standards, tritiated water, tritium

## Abstract

As part of the preparation and calibration of three new National Institute of Standards and Technology (NIST) tritiated-water radioactivity Standard Reference Materials (SRMs), the massic-activity (activity of the sample divided by the mass of the sample) ratios of all of the available NBS/NIST tritiated-water SRMs issued between 1954 and 1999 were measured using liquid-scintillation (LS) counting. Four of the tritiated-water standards (SRMs 4361, 4926B, 4927C, and 4927D) were not available for measurement. All of the other tritiated-water standards (SRMs 4361B, 4361C, 4926, 4926C, 4926D, 4926E, 4927, 4927B, 4927E, and 4927F) were available, having been stored in flame-sealed glass ampoules. Where possible, massic-activity ratios measured by liquid-scintillation counting are compared with massic-activity ratios calculated from gravimetric dilution factors. The agreement is well within the stated uncertainties. For two of the tritiated-water standards that were not available for measurement (SRMs 4361 and 4926B), massic-activity ratios calculated from gravimetric dilution factors are available.

## 1. Introduction

The National Institute of Standards and Technology (NIST), previously the National Bureau of Standards (NBS), has issued solution standards of tritiated water since 1954. A chronology is given in Table 1. The original solutions, NBS Standard No. 4926 and NBS Standard No. 4927, were calibrated using calorimetry [1]. In 1961, the massic activity (activity of the sample divided by the mass of the sample) of NBS Standard No. 4927 was measured in the (then) new NBS length-compensated internal gas proportional counters [2] and a revised certificate was issued. The massic activities of NBS Standard No. 4927 and of the new Standard Reference Material (SRM) 4927B were measured in 1978 using the same counters [3]. The massic activities of SRM 4927E and of NBS Standard No. 4927 were measured in 1998 and 1999, respectively, in the same counters [4].

As part of the preparation and calibration of three new NIST tritiated-water radioactivity SRMs, the massic-activity ratios of all of the available NBS/NIST tritiated-water SRMs issued between 1954 and 1999, and of samples of the Level 1 and Level 3 tritiated-water standards produced by the National Physical Laboratory (NPL) in 1996, were measured using liquid-scintillation (LS) counting. The results for the NPL samples are reported elsewhere [4]. Four of the tritiated-water standards (SRMs 4361, 4926B, 4927C, and 4927D) were not available for measurement. All of the other tritiated-water standards (SRMs 4361B, 4361C, 4926, 4926C, 4926D, 4926E, 4927, 4927B, 4927E, and 4927F) were available, having been stored in flame-sealed glass ampoules. Where possible, massic-activity ratios measured by liquid-scintillation counting are compared with massic-activity ratios calculated from gravimetric dilution factors. The agreement is well within the stated uncertainties. For two of the tritiated-water standards that were not available for measurement (SRMS 4361 and 4926B), massic-activity ratios calculated from gravimetric dilution factors are available.

## 2. Liquid-Scintillation Measurements

As part of the preparation and calibration of a new series of tritiated-water SRMs (4361C, 4926E, and 4927F), five batches of liquid-scintillation samples were prepared during 1998 and 1999. The first batch compared the massic activities of the new medium-level (SRM 4926E) and the new and old low-level (4361C and 4361B) tritiated-water SRMs. The second, third, fourth, and fifth batches compared the massic activities of all of the available high-level (4927, 4927B, 4927E, and 4927F) and medium-level (4926, 4926C, 4926D, and 4926E) tritiated-water SRMs, and of several samples of the Level 1 and Level 3 tritiated-water standards produced by NPL in 1996. For most of the SRMs, several ampoules of the SRM were sampled. Several independent dilutions of each high-level SRM or standard were made so that all of the LS vials had approximately the same count rate. This minimized any uncertainty due to dead time variation.

For each batch, four LS vials were prepared from each sample (ampoule or dilution) of each SRM or NPL standard. Each LS vial consisted of 10 mL of Beckman ReadySafe cocktail in a Beckman 18 mL polyethylene vial^1^. Each of the four LS vials was quenched with a different (but reproducible) amount of distilled water. The range of quench corresponded to a change in average pulse height of about 20 %. Approximately the same mass of tritiated water was weighed into each of the LS vials. The LS vials were then counted in one of two commercial LS counters, a Beckman LS7800 or a Packard 2500TR. Both counters use an external gamma-ray source to determine a quench indicating parameter (*QIP*). The counting window was set for tritium (0 keV to 18.6 keV). One counting cycle consisted of sequentially counting each LS vial in a given batch for 15 min. One run consisted of this counting cycle repeated 10 times. The data for each run were analyzed separately. The LS vials were then counted for 10 cycles in the other LS counter. This was repeated twice, so that the total count time for each LS vial was 600 minutes (2 counters times 2 runs per counter times 10 cycles per run times 15 min. per cycle) over a period of about 4 weeks.

For each run, a second-order polynomial (*y* = massic count rate, *x* = *QIP*) was fitted to the data (typically 40 points) for each set of four LS vials using a least-squares technique. Three values of the *QIP* were selected (low, middle, and high) that could be used for all of the data in a given run. The massic count rate for each sample, at each of the three *QIP* values, was then calculated using the polynomial. For any two samples, three ratios of the massic count rate (one at each of the selected *QIP* values) were obtained. The ratio of massic activities of any two samples in a given run was taken to be the average of these three massic count rate ratios. The standard deviation of the mean of the three ratios was used as an indication of the uncertainty associated with the ratio.

The final ratio of the massic activities of any two samples was taken to be the average of the ratios from all of the runs in which the two samples were intercompared. Because the liquid-scintillation samples had been so carefully matched with regard to other parameters, the relative standard deviation of the mean was used as the best estimate of the relative standard uncertainty associated with the final ratio.

## 3. Discussion of Results

Table 2 gives the massic-activity ratios for the high-level and medium-level SRMs. Table 3 gives the massic-activity ratios for the medium-level and low-level SRMs. For some of the SRMs, massic-activity ratios calculated from gravimetric dilution factors are available. These are given in Table 4 and, where possible, are compared with the massic-activity ratios measured by liquid-scintillation counting. It can be seen that the agreement is very good. This suggests that the massic activity ratios for SRM 4927 / SRM 4926B and SRM 4926 / SRM 4361, calculated from the gravimetric dilution factors, are probably also reliable to within the estimated uncertainty.

Using the NIST length-compensated internal gas proportional counters, the massic activity of SRM 4927E was determined to be 370 060 Bq·g^−1^ as of 1200 EST, 3 September 1998, with a relative expanded uncertainty (coverage factor *k* = 2) of 0.70 % [4]. Using the massic activity value for SRM 4927E and the massic-activity ratios in the tables, one can calculate the massic activity of any of the other SRMs (except SRMs 4927C and 4927D). Where a different reference time is desired for the massic activity, the recommended half-life is (4500 ± 8) d, where 8 d corresponds to one standard uncertainty [5].

## Figures and Tables

**Table 1:** Chronology of the NBS/NIST tritiated-water standards issued between 1954 and 1999

Year Issued	High-level standard	Medium-level standard	Low-level standard	Comments
1954	NBS 4927	NBS 4926		Calibrated by calorimetry. NBS 4926 is a quantitative dilution of NBS 4927.
1961	NBS 4927	NBS 4926		Recalibrated by gas counting. Revised certificate issued.
1965				New standards are now called NBS Standard Reference Materials (SRMs).
1977		SRM 4926B		SRM 4926B is a quantitative dilution of NBS 4927.
1979	SRM 4927B	SRM 4926C		Calibrated by gas counting. SRM 4926C is a quantitative dilution of SRM 4927B.
1981			SRM 4361	SRM 4361 is a quantitative dilution of NBS 4926.
1985	SRM 4927C			a
1987			SRM 4361B	SRM 4361B is a quantitative dilution of SRM 4926C.
1989	SRM 4927D	SRM 4926D		SRM 4926D is *not* a quantitative dilution of SRM 4927D.^a^
1991	SRM 4927E			a
1999	SRM 4927F	SRM 4926E	SRM 4361C	Calibrated by gas counting. SRM 4926E is a quantitative dilution of SRM 4927F. SRM 4361C is a quantitative dilution of SRM 4926E.

a The massic activities on the Certificates for SRMs 4927C, 4927D, 4926D, and 4927E were obtained using liquid-scintillation counting to determine the massic-activity ratios to SRM 4927B.

**Table 2:** Massic-activity ratios measured by liquid-scintillation counting for NBS/NIST tritiated-water standards issued between 1954 and 1999: high-level and medium-level standards. Each ratio is the massic activity of the SRM listed at the top of the table divided by the massic activity of the SRM listed on the left side of the table. The relative standard uncertainties of the ratios are shown as percentages. The number of sets of liquid-scintillation measurements are shown in parentheses

Ratio (Top/Side)	SRM 4927F	SRM 4927E	SRM 4927D	SRM 4927C	SRM 4927B	SRM 4927
SRM 4927F	1.00000					
SRM 4927E	1.71524 0.052 % (7)	1.00000				
SRM 4927D			1.00000			
SRM 4927C				1.00000		
SRM 4927B	2.84583 0.064 % (7)	1.65892 0.036 % (11)			1.00000	
SRM 4927	5.75385 0.056 % (4)	3.35603 0.046 % (8)			2.02372 0.016 % (12)	1.00000
SRM 4926E	125.959^a^ 0.041 % (7)	73.4585 0.013 % (7)			44.2898 0.071 % (11)	21.9070 0.005 % (8)
SRM 4926D	369.546 0.086 % (7)	215.450 0.034 % (7)			129.939 0.107 % (11)	64.2939 0.020 % (8)
SRM 4926C	576.758 0.066 % (4)	336.082 0.049 % (4)			202.760^a^ 0.047 % (8)	100.184 0.055 % (8)
SRM 4926B						a
SRM 4926	576.398 0.049 % (4)	336.261 0.058 % (8)			202.778^a^ 0.029 % (12)	100.201^a^ 0.023 % (12)

a The massic-activity ratio calculated from the gravimetric dilution factor is available; see Table 4.

**Table 3:** Massic-activity ratios measured by liquid-scintillation counting for NBS/NIST tritiated-water standards issued between 1954 and 1999: medium-level and low-level standards. Each ratio is the massic activity of the SRM listed at the top of the table divided by the massic activity of the SRM listed on the left side of the table. The relative standard uncertainties of the ratios are shown as percentages. The number of sets of liquid-scintillation measurements are shown in parentheses

Ratio (Top/Side)	SRM 4926E	SRM 4926D	SRM 4926C	SRM 4926B	SRM 4926	SRM 4361C	SRM 4361B
SRM 4926E	1.00000						
SRM 4926D	2.93382 0.038 % (11)	1.00000					
SRM 4926C	4.57316 0.050 % (8)	1.55823 0.074 % (8)	1.00000				
SRM 4926B				1.00000			
SRM 4926	4.57357 0.022 % (8)	1.55837 0.003 % (8)	1.00019 0.042 % (8)		1.00000		
SRM 4361C	2504.65^a^ 0.17 % (4)					1.00000	
SRM 4361B	8404.44^b^ 0.58 % (4)		a			3.35548^b^ 0.53 % (4)	1.00000
SRM 4361					a		

a The massic-activity ratio calculated from the gravimetric dilution factor is available; see Table 4.

b The uncertainty associated with this measurement is much larger than for most of the liquid-scintillation measurements because of the very low massic activity of SRM 4361B at the time of measurement.

**Table 4:** Massic-activity ratios measured by liquid-scintillation counting compared to massic-activity ratios calculated from gravimetric dilution factors. The relative standard uncertainties of the ratios are shown as percentages. The number of sets of liquid-scintillation measurements are shown in parentheses

Massic-activity Ratio	From the dilution factor	From liquid-scintillation	Difference
SRM 4927F / SRM 4926E	125.880 ≈0.05 %	125.959 0.041 % (7)	+0.06 %
SRM 4927B / SRM 4926C	202.905 ≈0.05 %	202.760 0.047 % (8)	−0.07 %
SRM 4927 / SRM 4926B	100.236 ≈0.06 %		
SRM 4927 / SRM 4926	100.148 ≈0.05 %	100.201 0.023 % (12)	+0.05 %
SRM 4926E / SRM 4361C	2507.56 ≈0.12 %	2504.65 0.17 % (4)	−0.12 %
SRM 4926C / SRM 4361B	1837.54 ≈0.15 %	1837.78^a^ 0.58 % (4)	+0.01 %
SRM 4926 / SRM 4361	2596.08 ≈0.20 %		

a Calculated as (SRM 4926E / SRM 4361B) / (SRM 4926E / SRM 4926C), using the massic-activity ratios given in Table 3.

## Footnotes

1 Certain commercial equipment, instruments, or materials are identified in this paper to foster understanding. Such identification does not imply recommendation or endorsement by the National Institute of Standards and Technology, nor does it imply that the materials or equipment identified are necessarily the best available for the purpose.
